# Inhibition of COX-2 expression by topical diclofenac enhanced radiation sensitivity via enhancement of TRAIL in human prostate adenocarcinoma xenograft model

**DOI:** 10.1186/1471-2490-13-1

**Published:** 2013-01-05

**Authors:** Takeshi Inoue, Satoshi Anai, Sayuri Onishi, Makito Miyake, Nobumichi Tanaka, Akihide Hirayama, Kiyohide Fujimoto, Yoshihiko Hirao

**Affiliations:** 1 Department of Urology, Nara Medical University, Nara, Japan

**Keywords:** Prostate cancer, Radiation therapy, COX-2, TRAIL, Apoptosis, Topical therapy, Radiosensitizer, Diclofenac, Radioresistance

## Abstract

**Background:**

COX-2 inhibitors have an antitumor potential and have been verified by many researchers. Treatment of cancer cells with external stressors such as irradiation can stimulate the over-expression of COX-2 and possibly confer radiation resistance. In this study, we tested if topical diclofenac, which inhibits both COX-1 and COX-2, administration rendered prostate tumor cells sensitize to the effects of radiation.

**Methods:**

LNCaP-COX-2 and LNCaP-Neo cells were treated with 0 to 1000 μM diclofenac. Next, a clonogenic assay was performed in which cells were subjected to irradiation (0 to 4 Gy) with or without diclofenac. COX-2 expression and other relevant molecules were measured by real-time PCR and immunohistochemistry after irradiation and diclofenac treatment. In addition, we assessed the tumor volumes of xenograft LNCaP-COX-2 cells treated with topical diclofenac with or without radiation therapy (RT).

**Results:**

LNCaP-COX-2 and LNCaP-Neo cell lines experienced cytotoxic effects of diclofenac in a dose related manner. Clonogenic assays demonstrated that LNCaP-COX-2 cells were significantly more resistant to RT than LNCaP-Neo cells. Furthermore, the addition of diclofenac sensitized LNCaP-COX-2 not but LNCaP-Neo cells to the cytocidal effects of radiation. In LNCaP-COX-2 cells, diclofenac enhanced radiation-induced apoptosis compared with RT alone. This phenomenon might be attributed to enhancement of RT-induced TRAIL expression as demonstrated by real-time PCR analysis. Lastly, tumor volumes of LNCaP-COX-2 cells xenograft treated with diclofenac or RT alone was >4-fold higher than in mice treated with combined diclofenac and radiation (p<0.05).

**Conclusions:**

These *in vitro* and *in vivo* findings suggest that conventional COX inhibitor, diclofenac enhances the effect of RT on prostate cancer cells that express COX-2. Thus, diclofenac may have potential as radiosensitizer for treatment of prostate cancer.

## Background

Cyclooxygenase (COX; prostaglandin G/H synthase, EC 1.14.99.1) is a key enzyme that catalyzes the conversion of arachidonic acid to prostaglandins (PGs) and other prostanoids.

Two isoforms of COX have been identified, COX-1 and COX-2 [1]. Recently, a splicing variant of COX-1 was identified. Alternative splicing generates four different mRNA variants derived from the COX-1 gene - COX-1, COX-3, and two partial COX-1s (PCOX-1 proteins). COX-3 and one of the PCOX-1 proteins (PCOX-1a) are made from the COX-1 gene but retain intron 1 in their mRNAs [2,3]. COX-1, constitutively expressed in almost tissues, is required for homeostatic functions, whereas COX-2, an inducible enzyme, is up-regulated by growth factors, tumor promoters, oncogenes and carcinogens [1,4-6].

Of the two isoforms, COX-2 is consistently up-regulated in a number of cancers, including esophagus [7], stomach [8], colon [9], lung [10], pancreas [11], head and neck [12], and prostate [13]. In addition, COX-2 expression is associated with aggressive tumor behavior, worse prognosis and the development of metastatic disease [14].

On immunohistochemical (IHC) staining of prostate, the expression of COX-2 protein in prostate cancer (PCa) was significantly higher than that in benign prostatic hyperplasia (BPH) and normal prostate, while the expression of COX-1 protein was not significantly different between BPH and PCa. Furthermore, the intensity of COX-2 expression increased with increasing tumor grade [13,15,16].

Clinical, and animal studies indicate that the use of aspirin and other non-steroidal anti-inflammatory drugs (NSAIDs) can significantly reduce the risk of colorectal, breast, prostate, lung and esophageal cancer [4,17-19]. As for prostate cancer, recent epidemiological studies indicated that use of aspirin and other NSAIDs reduces the risk of prostate cancer by 20-30% [20].

A number of studies suggest that COX-2 expression is not only an early event in the carcinogenesis, but is required throughout the entire evolutionary process of cancer development and progression [18]. Furthermore, it has been proposed that COX-2 may regulate the expression of genes that contribute to tumor cell survival, aggressiveness, and angiogenesis. A number of previous studies have shown that PGs can act as potent radioresistant factors of some cancers, and their inhibition resulted in radiosensitization [21-24]. Therefore, COX-2, which induced PGs, is considered to be potential targets for anticancer therapy. Although COX is the only molecular target known of most NSAIDs, both COX-dependent and COX-independent mechanism in the apoptotic action of NSAIDs have been reported [25-28].

Diclofenac is a selective COX-1 and COX-2 inhibitor. The reason why we used this drug in this study was that diclofenac was a widely used non-steroidal anti-inflammatory drug and topical diclofenac was available.

In the present study, we investigated the role of COX-2 expression in radioresistance and the effect of diclofenac on the cell viability and radiosensitivity of prostate cancer cells *in vitro* experiments. Furthermore, we investigated the efficacy of treatment with topical diclofenac using diclofenac gel *in vivo* experiment.

## Results

### Effect of diclofenac on cell viability of prostate cancer cells

To evaluate the antitumor potential of diclofenac on cell viability and whether COX-2 expression contributes to cell proliferation, LNCaP-COX-2 cells and LNCaP-Neo were treated with various concentrations of diclofenac for 72 hours.

**Figure 1:**
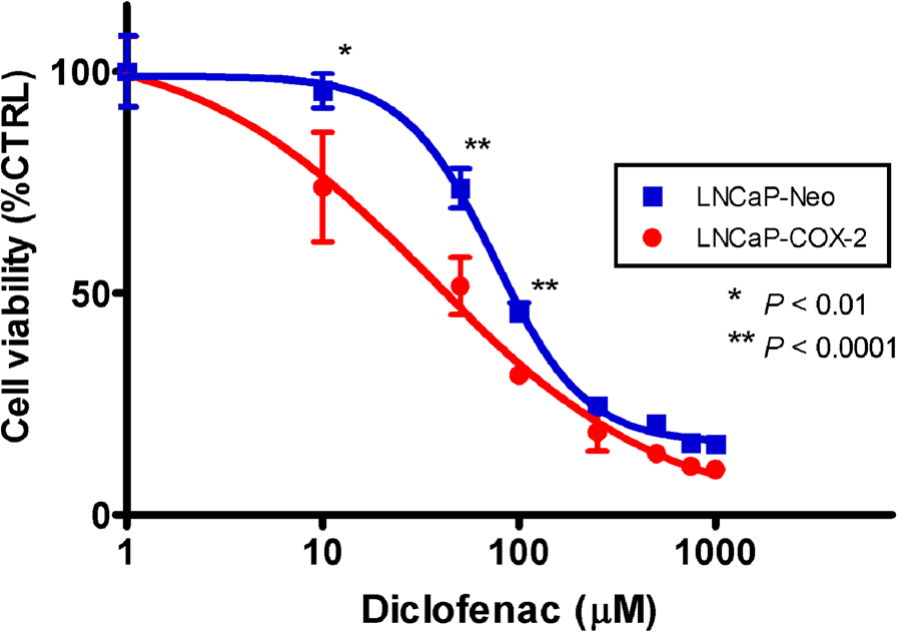
**Effects of diclofenac on LNCaP-COX-2 cells and LNCaP-Neo.** Cells were seeded in 96-well plate, and treated with indicated concentration of diclofenac for 72 hours. Cytotoxicity was determined by WST-8 assay. Diclofenac reduced cell viability in both LNCaP-COX-2 cells and LNCaP-Neo cells in a dose-dependent manner. LNCaP-COX-2 cells significantly exhibited higher sensitivity to diclofenac than LNCaP-Neo cells. Cell viability at 10 μM in LNCaP-COX-2 and LNCaP-Neo was 74.0% and 95.7% (*p* = 0.0094), 51.6% and 73.8% at 50 μM (*p* < 0.0001), 31.7% and 45.7% at 100 μM (*p* < 0.0001). Absorbance values were normalized to values for vehicle only treated cells (control), and the results are expressed as the mean ± SD of sextuplet samples.

Diclofenac reduced cell viability in both LNCaP-COX-2 cells and LNCaP-Neo in a dose-dependent manner (Figure 1). However, LNCaP-COX-2 cells significantly showed higher sensitivity to diclofenac than LNCaP-Neo. Cell viability at 50 μM in LNCaP-COX-2 and LNCaP-Neo was 51.6% and 73.8%, respectively (p < 0.0001). The diclofenac 50% inhibitory concentration (IC50) was calculated in LNCaP-COX-2 and LNCaP-Neo and found to be 42.2 μM and 91.6 μM, respectively. This data suggest that susceptibility to diclofenac may be attributed to the level of COX-2 expression.

### Effect of diclofenac on radiosensitivity of prostate cancer cells

**Figure 2:**
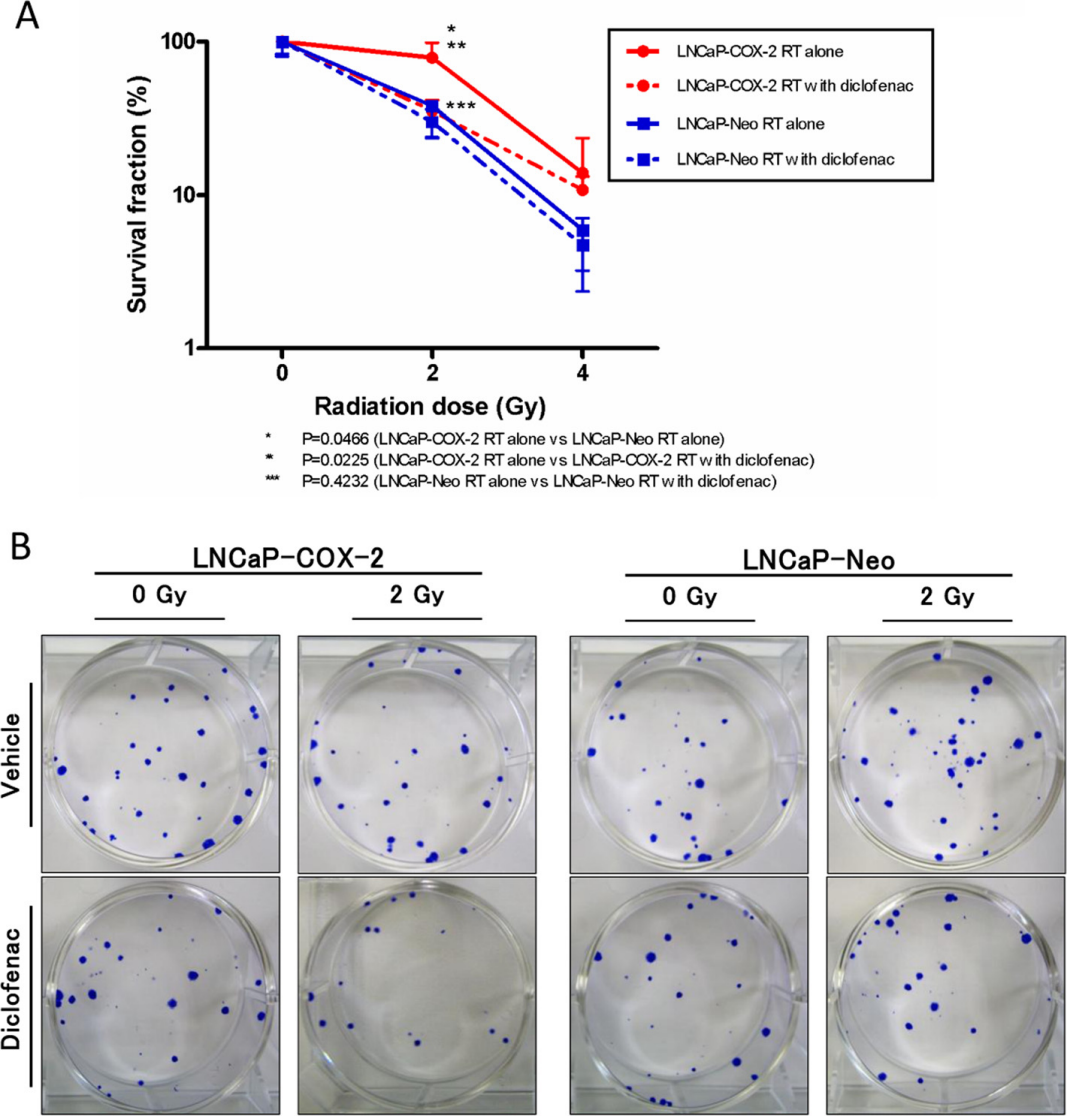
**Clonogenic assay for LNCaP-COX-2 and LNCaP-Neo treated with irradiation.** Clonogenic assay for LNCaP-COX-2 and LNCaP-Neo treated with irradiation (0, 2 or 4 Gy) with or without diclofenac (IC25). Cells were seeded in 6-well plate, and treated with or without diclofenac for 48 hours, irradiated, and allowed to form colonies. (**A**) Survival fractions for radiation and diclofenac were normalized by dividing by the survival fractions for untreated cells. LNCaP-COX-2 cells was more radioresistant than LNCaP-Neo cells (*p* = 0.0225). In LNCaP-COX-2 cells, diclofenac enhanced the effect of RT with significant differences between RT alone and RT with diclofenac, when cells were exposed to 2 Gy (*p* = 0.0466). (**B**) Clonogenic assay of LNCaP-COX-2 cells and LNCaP-Neo cells treated with RT (0, 2 or 4 Gy) and diclofenac (0 or IC25). Photographs of 6-well plates in a representative experiment are shown.

To determine the effect of diclofenac on radiosensitivity of prostate cancer cells, we next examined a clonogenic assay. LNCaP-COX-2 and LNCaP-Neo cells were treated with 10.9 μM and 46.7 μM (respective IC25 of cell lines) of diclofenac for 48 hours before irradiation (0, 2 or 4 Gy). The survival fraction of both LNCaP-COX-2 cells and LNCaP-Neo cells was decreased in a radiation dose-dependent manner (Figure 2A, 2B). The survival fractions at 2 Gy dose without diclofenac were 78.6% and 38.2% (p = 0.0466) for LNCaP-COX-2 and LNCaP-Neo, respectively. LNCaP-COX-2 cells were proved to be more radioresistant than LNCaP-Neo cells. Furthermore, diclofenac significantly sensitized the LNCaP-COX-2 cells to RT. However, this effect was not observed in LNCaP-Neo cells. In LNCaP-COX-2 cells, the survival fractions at 2 Gy dose were 78.6% and 35.5% for radiation alone and radiation with diclofenac, respectively (p = 0.0225), while the survival fractions were 38.2% and 30.0%, respectively in LNCaP-Neo (p = 0.4232). This data suggested that COX-2 overexpression protected LNCaP-COX-2 cells from the effect of irradiation and that inhibition of COX-2 sensitized to RT.

### Diclofenac suppressed RT-induced COX-2 up-regulation and enhanced the induction of TRAIL

**Figure 3:**
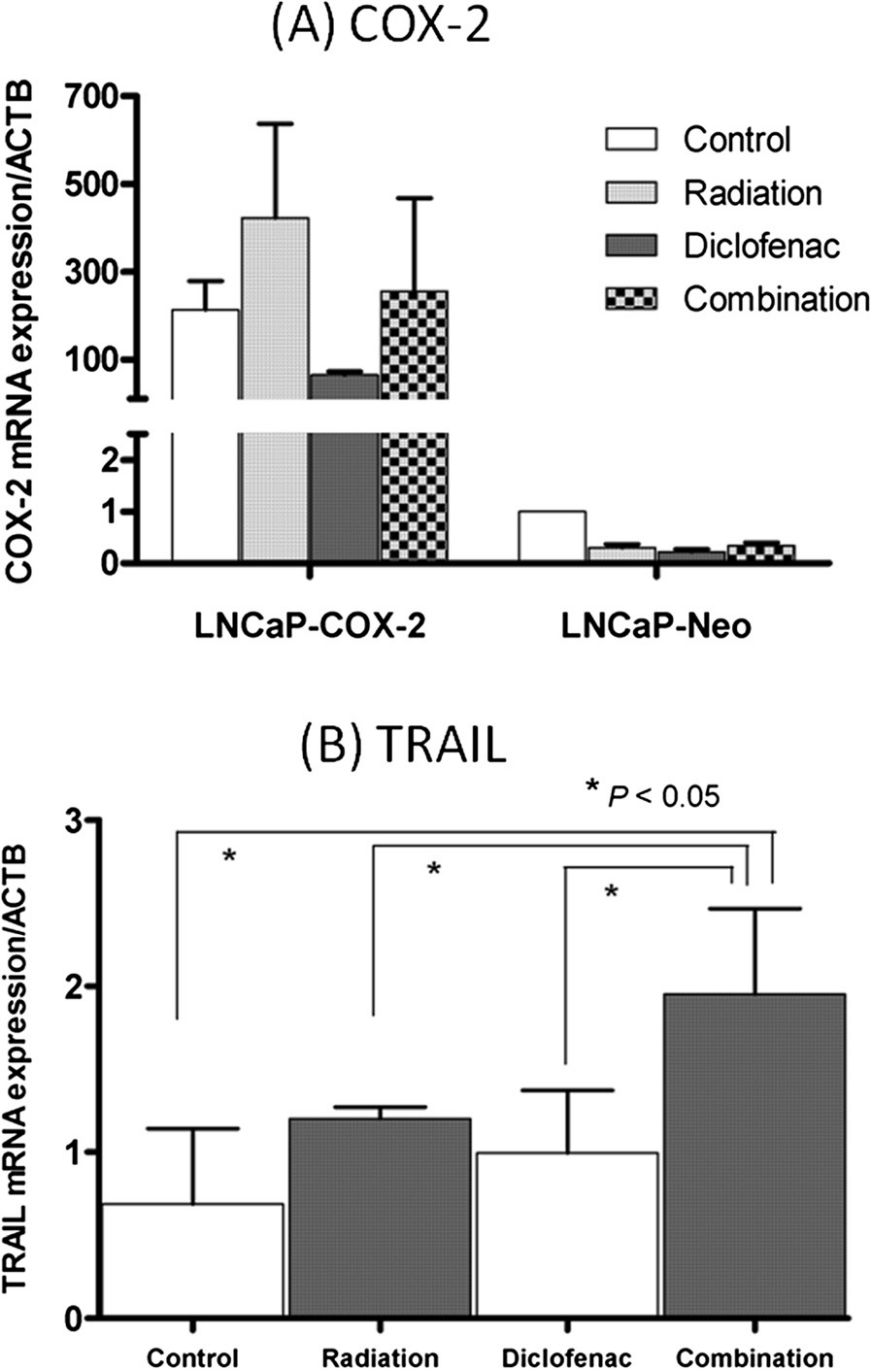
**Real-time PCR analysis for COX-2 (A) and TRAIL (B).** RT induced COX-2 up-regulation, and diclofenac inhibited RT-induced COX-2 up-regulation. Combination of radiation and diclofenac significantly enhanced the effect of RT on induction of TRAIL (*p* < 0.05).

Next, we set out to assess the molecular profile of treated cells using real-time PCR analysis. We focused on expression of COX-2 and TRAIL. We investigated the release of TRAIL in response to RT since TRAIL is a most common RT-inducible cytokine [29-31]. In LNCaP-COX-2 cells, RT induced COX-2 up-regulation but diclofenac inhibited RT-induced COX-2 up-regulation (Figure 3A). Furthermore, RT induced TRAIL, and combination with diclofenac enhanced the induction of TRAIL (Figure 3B). Thus, diclofenac made LNCaP-COX-2 cells more sensitive to apoptosis through induction of TRAIL.

### Combination of diclofenac and radiation induced apoptosis in LNCaP-COX-2 cells

**Figure 4:**
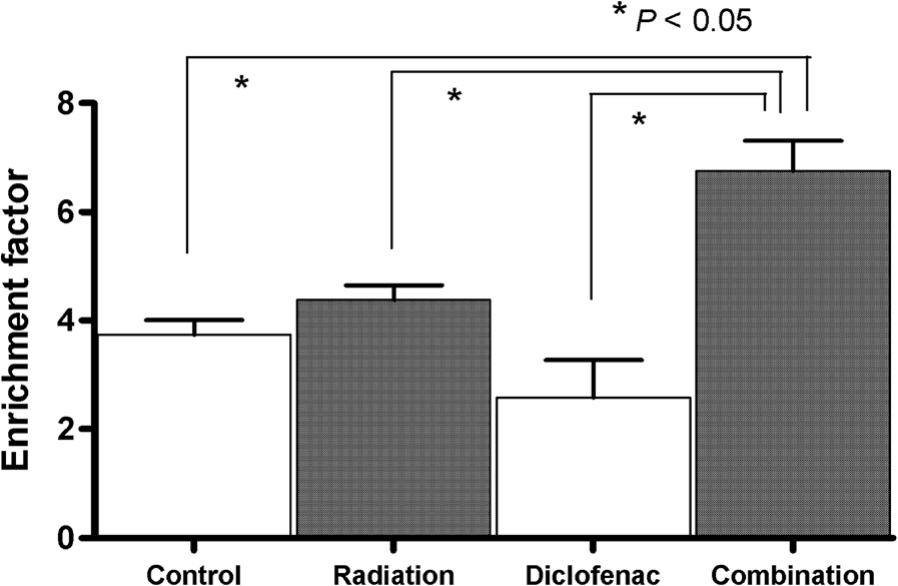
**Combined treatment with radiation and diclofenac increased an induction of apoptosis compared with either alone.** Cells were incubated for 24 hours, and treated with diclofenac (IC25) for 48 hours, then exposed to radiation (0, 2 Gy). Apoptosis induction was quantified using Cell Death Detection ELISA kit.

According to the results of real-time PCR analysis, TRAIL mRNA expression was found to induce by diclofenac. Consequently, to determine whether the combination of diclofenac and radiation was related with apoptosis, cells were treated with diclofenac (IC50) for 3 hours, thereafter irradiated with 2 Gy. After incubation for 3 hours, the cells were harvested to examine apoptosis using Cell Death Detection ELISA^Plus^ kit according manufacturer’s instructions. Apoptosis was not significantly induced in cells treated with either diclofenac alone or radiation alone compared with untreated cells. However, combined treatment of diclofenac and irradiation was significantly induced apoptosis much than untreated cells (Figure 4). This apoptosis was responsible for induction of TRAIL that induced by combined treatment.

### Topical treatment with diclofenac reduced tumor growth and enhanced the effect of RT *in vivo* xenograft mouse model, and suppressed both COX-2 and Ki-67 expression

**Figure 5:**
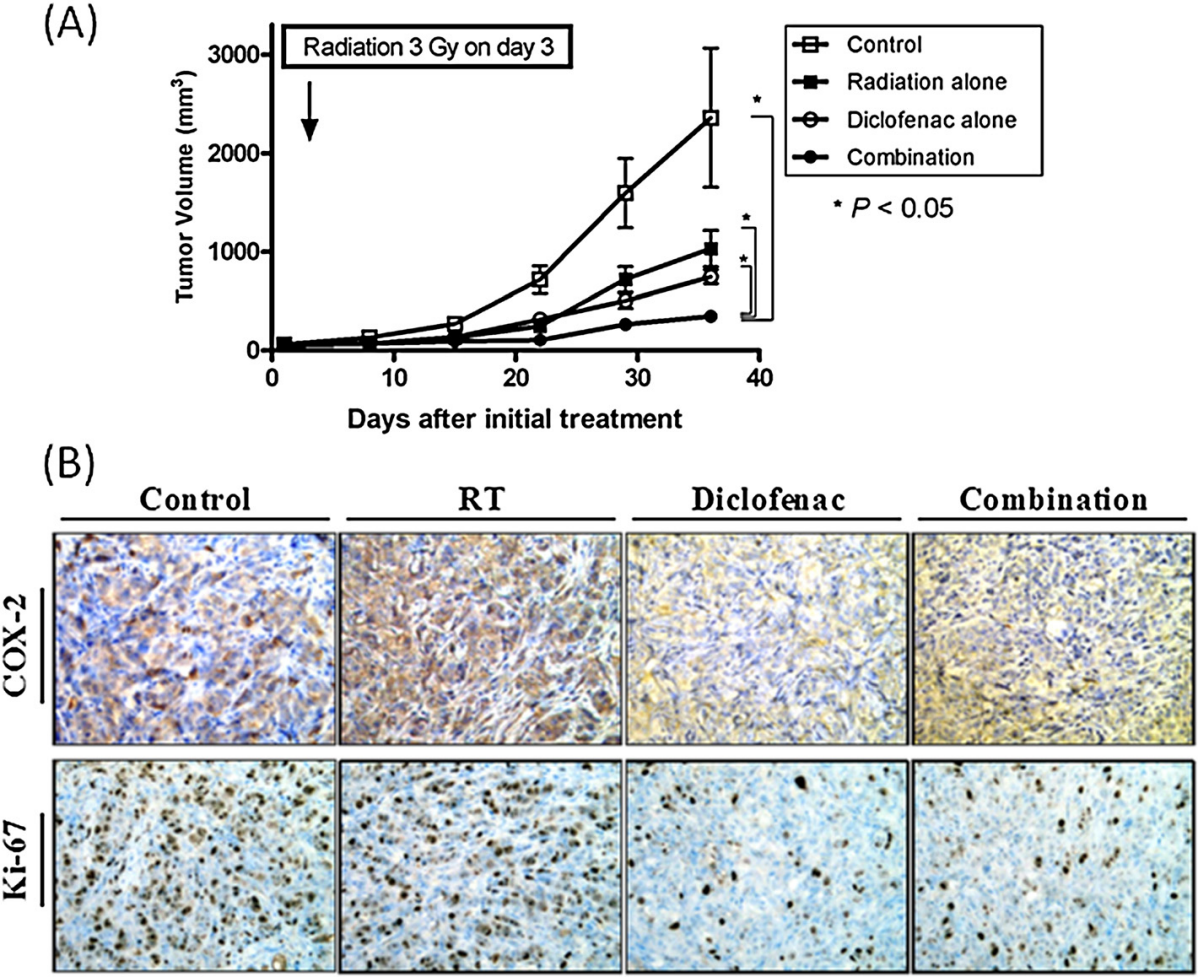
**Diclofenac induced the tumor growth delay and enhanced the effect of RT in xenograft mouse model of LNCaP-COX-2.** The xenograft mice bearing LNCaP-COX-2 tumor were treated with topical administration of diclofenac gel onto mice skin with cotton swab once a day (day 1–36). The tumors in RT alone group and combination group were exposed to IR with a single dose of 3 Gy on day 3. Tumor volume was measured with calipers and calculated as 0.52LW^2^. (**A**) Tumor volume was significantly reduced in combination group. (**B**) Immunohistochemical staining for COX-2 and Ki-67 was conducted. The expression of COX-2 decreased in diclofenac group and combination group compared with control group, while the expression of COX-2 increased in RT group. The expression of Ki-67 significantly decreased in diclofenac group and combination group. Original magnification, x200.

To confirm radiosensitizing effect of diclofenac *in vivo*, we conducted animal experiments using xenograft mouse model of LNCaP-COX-2. The xenograft mice bearing LNCaP-COX-2 tumor were treated with topical administration of diclofenac gel onto mice skin with cotton swab once a day with or without irradiation (3 Gy) to the subcutaneous tumor on day 3. Treatment with diclofenac reduced tumor growth to 37.0% compared to control, whereas treatment with 3 Gy radiation reduced tumor growth to 57.0% compared to control. The mean relative tumor volumes in control and combined treatment (RT and diclofenac) groups on day 36 were 35.74 fold-change and 5.96, respectively (p < 0.05). In addition, combination therapy resulted in enhanced antitumor potential compared with diclofenac alone or radiation alone (Figure 5A). No weight loss was observed in any of the experimental groups (data not shown).

To investigate the cell proliferation status and level of COX-2 expression in the treated and control tumors, the expression of Ki-67 and COX-2 were analyzed with IHC staining. The expression of COX-2 decreased in diclofenac group and combination group compared with control group, while the expression of COX-2 increased in RT group. The expression of Ki-67, proliferative index (PI), significantly decreased in diclofenac group (PI: 0.21) and combination group (PI: 0.31) compared with control group (PI: 0.61) (Figure 5B).

## Discussion

PCa is the most common solid tumor in US males and the second leading cause of cancer related deaths [32]. A common treatment option for localized PCa is RT, which may possess similar survival rates at 5, 10, and 15-years to radical prostatectomy. Though radiation dose increase has associated with higher cancer control rates, high dose radiation can cause important side effects, such as urinary dysfunction, impotence, and rectal symptoms. Several reports have demonstrated that the addition of androgen deprivation to RT may improved the results of patients with intermediate- and high-risk PCa. However in high-risk patients treated with RT, 5-year recurrence free survival is approximately 50%.

Thus, new molecular targets for the enhancement of RT and regression of RT adverse event are needed for some patients with localized prostate cancer.

NSAIDs have been demonstrated to potentiate radiosensitivity of cancer cells, and recent studies reported the radiosensitizing effect of selective COX-2 inhibitors on tumor cells [23-25,33].

The cells overexpressing COX-2 tend to be resistant to apoptosis, and COX-2 inhibitors have been shown to induce apoptosis in these types of cells. Therefore, we hypothesized that the COX inhibitor, diclofenac could enhance the effect of radiation on prostate cancer cells that constitutively express COX-2.

Although many of recent studies reported about the relation between COX inhibitor and radiosensitizing effect, conducted chiefly *in vitro* experiments study. We further attempted to show the potentiation of RT *in vivo* experiments.

This study is the first evidence that treatment with diclofenac increased the radiosensitivity of prostate cancer cells by suppression of COX-2 up-regulation and induction of TRAIL *in vitro*, and that topical treatment with diclofenac gel enhanced antitumor potential of RT *in vivo*.

The underlying mechanism responsible for the antitumor effect of COX inhibitor has not been clearly elucidated, although several possibilities have been proposed, i.e. modulation of angiogenesis, regulation of cell cycle, and reverse of PGs-induced immunosuppression [9]. PGs, which were produced by COX-2, have been proposed to promote the proliferation and metastasis of cancer cells and secondarily encourage the growth of cancer cells by immunosuppression. Furthermore, PGE1 and PGE2 are known to induce angiogenesis [9,18]. Milas and his colleagues demonstrated that the NSAID indomethacin prolongs tumor growth delay and increases the tumor cure rate after radiotherapy [22,24]. It has been proposed that NSAIDs increased tumor radiation response by lowering the level of PGs in the tumor [23]. Based on recent findings that PGs and COX-2 are radioprotective factors, it is reasonable to assume that diclofenac inhibited tumor cell proliferation and increased tumor radiosensitivity by reducing tumor-producing PGs [34,35]. Recent experiments using selective or not selective COX-2 inhibitor, including celecoxib and NS-398, demonstrated that COX-2 overexpression might be responsible for radioresistance [6,25], and that suppression of COX-2 overexpression render cells susceptible to RT.

NSAIDs have been demonstrated to potentiate the effect of RT on cancer cells *in vitro* and *in vivo*, however, no study has reported on the efficacy of topical diclofenac gel.

In this study, we investigated the antitumor potential and the radiosensitizing effect of diclofenac on prostate cancer cells. In LNCaP-COX-2 cells, diclofenac significantly reduced the cell viability and sensitized to RT. COX-2 overexpression is responsible for radioresistance in clonogenic assay. LNCaP-COX-2 cells were more susceptible to diclofenac than LNCaP-neo cells. These findings suggested that LNCaP-COX-2 might be addictive to COX-2 than LNCaP-Neo cells, and that COX-2 might be one of survival factors for LNCaP-COX-2 cells. We first showed that the combination of RT and topical treatment with diclofenac gel significantly induced the tumor growth delay than either treatment alone in prostate cancer tumor *in vivo*. The radiosensitizing effect of diclofenac was not observed in LNCaP-Neo cells that lacked COX-2 expression.

In real-time PCR analysis, RT induced TRAIL and combination with diclofenac enhanced the RT-induced TRAIL.

We first suggested that additional aspects of the COX inhibitor, diclofenac-induced potentiation of tumor radioresponse were caused by the induction of TRAIL.

TRAIL is an identified member of the TNF ligand family that can induce a rapid caspase-dependent apoptosis with high specificity for malignant cells.

Recent study has been shown that significant release of TNF-related apoptosis inducing ligand was observed in response to ionizing radiation(IR) in lung cancer cells [36], and that IR up-regulates TRAIL-Receptor surface expression [37]. TNF-α and TRAIL are directly involved in apoptosis and are induced by IR [38]. Irradiation induced release of tumor necrosis factor-α, or TRAIL [36].

Our findings that RT significantly induced TRAIL in real-time PCR analysis are consistent with these results. In addition, combination of RT and diclofenac significantly increased the induction of TRAIL compared with either alone. The enhancement of TRAIL can explained the observed increase of apoptosis.

Apoptosis is controlled via two major pathways, including one that originates at the cell membrane and another that involves the mitochondria. The membrane death receptor (DR) pathway involves DRs such as Fas, TNF-R1, DR-3, DR-4, and DR-5, that are activates by their respective ligands and engages the intracellular apoptotic machinery. In addition, caspase pathway is involved in TRAIL-induced cell death, and IR-induced TRAIL contributed to cell death via increase of caspase-8, caspase-9, and caspase-3 activation. COX-2 expression has shown to significantly attenuate TRAIL-induced caspase-8, caspase-9, and caspase-3 [31]. NSAIDs, sulindac sulfide, increased both DR-4 and DR-5 mRNA levels. Modulation of the level of DR-5 regulated the apoptotic response to TRAIL [31]. RT induced TRAIL in both LNCaP-COX-2 and LNCaP-Neo cells (data not shown) in real time PCR analysis. COX-2 overexpression was shown to attenuate the effect of TRAIL-induced apoptosis and reduce Fas-mediated apoptosis [31,39].

## Conclusions

This study reveals that COX-2 is a surviving factor associated with radiation resistance. Diclofenac sodium suppressed COX-2, induced by irradiation, which rendered cells resistant to RT. In addition, combination therapy of RT plus diclofenac enhanced the expression of TRAIL, and therefore induced apoptosis in prostate cancer cells compared with RT or diclofenac alone. These findings suggest that diclofenac may be the effective radiosensitizer against human prostate cancer over-expressed COX-2.

## Methods

### Chemical compounds and reagents

A selective COX-1 and COX-2 inhibitor, diclofenac sodium (diclofenac), was purchased from Sigma-Aldrich (St Louis, MO, USA). For *in vitro* studies, diclofenac was dissolved in distilled water at a stock concentration of 50 mM and this stock was diluted in media just before use. For *in vivo* studies, diclofenac sodium 1% topical gel was purchased from Novartis Pharma AG (Basel, Switzerland).

### Cell culture and transfection

The human prostate cancer cell line LNCaP was purchased from American Type Culture Collection (Manassas, VA, USA) and was maintained in RPMI 1640 supplemented with 10% fetal bovine serum, penicillin-streptomycin (100 units/ml and 10 μg/ml) in a standard incubator at 37°C with 5% CO_2_.

LNCaP-COX-2 cells, stably transfected to overexpress COX-2, and LNCaP-Neo cells as the control were kindly provided by Dr. Astushi Mizokami (Kanazawa, Japan).

### Irradiation procedures

To determine sensitivity to irradiation, plated prostate cells were exposed to radiation dose using a MBR-1520R X-ray unit (Hitachi Medical Co., Tokyo, Japan). The irradiation for *in vivo* experiments was described later.

### *In vitro* cell viability assay

Cells were seeded in a 96-well plate at a density of 2,000 cells per well in growth media and incubated for 24 hours. They were treated with various concentrations of diclofenac sodium ranging from 0–1000 μM. After incubating the plates for 48 hours, cell viability was determined using a Cell Counting Kit-8 (Dojindo Labor-atories, Kumamoto, Japan) according to the manufacturer’s instructions. The viability index was expressed by relative value to the untreated cells. Each assay was performed in sextuplet. Dose response curves were generated. The data are expressed as means ± standard deviations (SD).

### Clonogenic assay

Cells were seeded in a 6-well plate at a density of 10 × 10^4^ cells per well in growth medium, and incubated for 24 hours. Then the cells were treated with 25% inhibitory concentration (IC25) of diclofenac sodium for 48 hours and the plates were exposed to 0–4 Gy radiation. Immediately after irradiation, the cells were trypsinized, serial diluted and seeded into 6-well plates. The plates were incubated for 14 days to allow colony formation. The colonies were stained with 0.25% crystal violet in ethanol and counted by eye with a cutoff of 50 viable cells. The survival fraction was calculated relative to non-irradiated cells. Data were shown as means ± SD.

### Real-time PCR analysis

Total RNA was extracted from cells using a QIAamp RNA Blood Mini kit according the manufacturer’s instructions (Qiagen, Valencia, CA, USA). RNA concentrations were determined by UV spectrophotometer, and 2 μg of total RNA was reverse transcribed into cDNA using the High Capacity cDNA Reverse Transcription Kit (Applied Biosystems, Foster City, CA, USA). Real-time PCR was performed on a StepOne Plus^TM^ Real-time PCR system (Applied Biosystems) with Fast SYBR Green Master Mix (Applied Biosystems). The PCR primer used in this study were: *ACTB*   forward   5^′^-CTGGAACGGTGAAGCTGACA-3^′^ and reverse 5^′^-CGGCCACATTGTGAACTTTG-3^′^,  *COX-2*   forward 5^′^-TGCATTCTTTGCCCAGCACT-3^′^  and reverse 5^′^-AAAGGCGCAGTTTACGCTGT-3^′^, *TRAIL*  forward  5^′^-GAAGCAACACATTGTCTTCTCCAA-3^′^   and reverse 5^′^-TTGCTCAGGAATGAATGCCC-3^′^.

The results were analyzed with the StepOne Software version 2.0 (Applied Biosystems), using β-actin gene expression as an internal control.

### Apoptosis measurement with cell death detection ELISA^Plus^ assay

The Cell Death Detection ELISA^Plus^ kit (Roche Molecular Biochemicals, Mannheim, Germany) was used to measure DNA fragmentation as a marker for apoptosis according to the manufacturer’s instructions.

Briefly, cells were seeded in 96-well plate at a density of 2,000 cells per well, and incubated for 24 hours. Consequently, the cells were treated with diclofenac sodium concentration (IC25) for 48 hours and the plates were exposed to 0–2 Gy radiation.

Cytoplasmic fractions of control and treated cells were transferred into streptavidin-coated 96-well plates and incubated with biotinylated mouse anti-histone antibody and peroxidase-conjugated mouse anti-DNA antibody at room temperature for 2 hours. The rate of apoptosis was determined by measurement at 405 nm using a microplate reader. The enrichment of mono- and oligonucleosomes released into the cytoplasm was calculated using the following formula: enrichment factor = absorbance of sample cells/absorbance of control cells.

### Xenograft mouse model and administration of diclofenac and IR

Male BALB/c nu/nu mice were purchased from Charles River Japan, Inc. (Yokohama, Japan). The mice were maintained under specific pathogen-free conditions and provided with sterile food and water, and used at 6–8 weeks of age. All animal protocols for this experiment were approved by the Animal Care Committee of the Nara Medical University in accordance with the policies established in the NIH Guide for the care and use of laboratory animals.

LNCaP-COX-2 cells (5 × 10^6^ cells) in 0.05 ml of RPMI medium, together with of 0.05 ml of Matrigel (Becton Dickson, Bedford, MA, USA), were injected subcutaneously into the bilateral flanks of each mouse. When the tumors reached 0.5 cm in diameter, the animals were divided randomly into four groups (control, diclofenac only, radiation only, diclofenac and radiation) (n=6 for each group) and treatment was initiated (Day 1).

In the treated groups, diclofenac sodium 1% gel was applied daily to the skin overlying the tumors with a cotton swab. At day 3, the mice were irradiated with 3 Gy dose using MBR-1520R X-ray unit. Only tumors treated with irradiation were left exposed. All other regions of the mice were shielded with lead. The tumor sizes and volumes were measured once a week with calipers, and the tumor volumes were calculated using the formula: 0.52 × (Length) × (Width)^2^.

### Immunohistochemical (IHC) staining

The resected tumors were fixed in 10% buffered formalin, embedded in paraffin, and sectioned (3-μm thickness). IHC staining for COX-2 and Ki-67 was conducted as follows: slides were deparaffinized and incubated for 5 min with 3% H_2_O_2_ to quench the endogenous peroxidase activity. Antigen retrieval was performed for 10 min in citrate buffer with autoclave treatment. Slides were incubated with primary antibody at 4°C overnight. Next, the tissue sections were incubated with mouse monoclonal anti-human COX-2 antibody (Santa Cruz, USA) and mouse monoclonal anti-human Ki-67 antibody (Dako, Glostrup, Denmark). Sections were then developed with diaminobenzidine (DAB), counterstained with hematoxylin, dehydrated, and mounted.

The Ki-67 proliferative index was defined as the proportion of Ki-67-positive tumor cells to the total tumor cells.

### Statistics

In *in vitro* experiments, differences between means were compared by the Student’s t-test. A *p*-value of 0.05 or less between groups was considered significant. Data were shown as means ± Standard deviations. In the analysis of *in vivo* experiments, statistical differences between the relative volumes in the treated and control groups were analyzed using Mann–Whitney U test (statistical tests were two-sided). A *p*-value of less than 0.05 was considered statistically significant.

## Competing interests

The authors declare that they have no competing interests.

## Authors’ contributions

TI and SA conceived the experiments. TI and SO conducted the experiments and analyzed the data together with SA. ST, AH, KF and YH supervised the experiments and the writing of the manuscript. All authors read and approved the final version of the manuscript.

## Pre-publication history

The pre-publication history for this paper can be accessed here:

http://www.biomedcentral.com/1471-2490/13/1/prepub
